# Lymph nodes ratio based nomogram predicts survival of resectable gastric cancer regardless of the number of examined lymph nodes

**DOI:** 10.18632/oncotarget.17276

**Published:** 2017-04-20

**Authors:** Shangxiang Chen, Huamin Rao, Jianjun Liu, Qirong Geng, Jing Guo, Pengfei Kong, Shun Li, Xuechao Liu, Xiaowei Sun, Youqing Zhan, Dazhi Xu

**Affiliations:** ^1^ State Key Laboratory of Oncology in South China, Collaborative Innovation Center for Cancer Medicine, Guangzhou, China; ^2^ Department of Abdominal Surgery, Jiangxi Cancer Hospital, Nanchang, China; ^3^ Department of Breast Surgery, Anhui Provincial Cancer Hospital, West Branch of Anhui Provincial Hospital, Hefei, China; ^4^ Department of Hematology Oncology, Sun Yat-sen University Cancer Center, Guangzhou, China; ^5^ Department of Gastric and Pancreatic Surgery, Sun Yat-sen University Cancer Center, Guangzhou, China

**Keywords:** nomogram, gastric cancer, curative resection, lymph nodes ratio, prognosis

## Abstract

To develop a nomogram to predict the prognosis of gastric cancer patients on the basis of metastatic lymph nodes ratio (mLNR), especially in the patients with total number of examined lymph nodes (TLN) less than 15. The nomogram was constructed based on a retrospective database that included 2,205 patients underwent curative resection in Cancer Center, Sun Yat-sen University (SYSUCC). Resectable gastric cancer (RGC) patients underwent curative resection before December 31, 2008 were assigned as the training set (n=1,470) and those between January 1, 2009 and December 31, 2012 were selected as the internal validation set (n=735). Additional external validations were also performed separately by an independent data set (n=602) from Jiangxi Provincial Cancer Hospital (JXCH) in Jiangxi, China and a data set (n=3,317) from the Surveillance, Epidemiology, and End Results (SEER) database. The Independent risk factors were identified by Multivariate Cox Regression. In the SYSUCC set, TNM (Tumor-node-metastasis) and TRM-based (Tumor-Positive Nodes Ratio-Metastasis) nomograms were constructed respectively. The TNM-based nomogram showed better discrimination than the AJCC-TNM staging system (C-index: 0.73 versus 0.69, p<0.01). When the mLNR was included in the nomogram, the C-index increased to 0.76. Furthermore, the C-index in the TRM-based nomogram was similar between TLN ≥16 (C-index: 0.77) and TLN ≤15 (C-index: 0.75). The discrimination was further ascertained by internal and external validations. We developed and validated a novel TRM-based nomogram that provided more accurate prediction of survival for gastric cancer patients who underwent curative resection, regardless of the number of examined lymph nodes.

## INTRODUCTION

Although the incidence of gastric cancer has declined recently, gastric cancer still remains one of the most common cancers. Nearly one million new gastric cancer cases are diagnosed every year [1]. Gastric cancer has been the second leading cause of cancer-related deaths all over the world [2], and has a 5-year survival of 28% or less [3]. Curative resection, as a standard surgery procedure, has been widely used in the treatment of gastric cancer [4].

However, prognosis of gastric cancer patients varies due to individual factors. Thus, a consensus standard is needed for prognostic prediction and individualized therapy scheduling. In 2010, the 7^th^ edition of the American Joint Committee on Cancer (AJCC) tumor–node-metastasis (TNM) staging system was published. It is a conventional method for prognostic prediction of gastric cancer [5]. Unfortunately, survival could usually vary from each other even in patients with the same AJCC stage. In fact, clinicopathological parameters like gender, age, tumor size, differentiation, and adjuvant chemotherapy were not involved in this system, and prognostic differences may be caused by these ignored but significant characteristics, which could affect the final survival status to some extent. Therefore, a more refined staging system considering both the tumor characteristics and host status is needed.

Nomogram, a better estimation of the prognosis, included aforementioned basic prognostic factors, has been developed for survival prediction in many other cancers [6–10]. Recently, several nomograms have also been established and validated in gastric cancer [11–15]. These prognostic models based on clinicopathological characteristics could predict the survival of gastric cancer patients more accurately compared with the AJCC TNM staging system.

However, metastatic lymph nodes ratio (mLNR), one of the most reliable predictors for curatively resected gastric cancer patients, has not been included in previous nomograms [16]. The mLNR, defined as the ratio of the metastatic lymph divided by the retrieved lymph nodes, showed significant superiority in minimizing ‘stage migration’ and has been demonstrated to be an independent prognostic factor in gastric cancer [17–20].

In the current study, we constructed a nomogram based on mLNR to predict the individualized survival of gastric cancer patients underwent curative resection. We also evaluated the significance of the nomogram in the patients with examined lymph nodes (TLN)≤15 for the first time. We supposed that the tumor–positive node ratio-metastasis (TRM)-based nomogram may work better in prognostic prediction when compared with the tumor–node-metastasis (TNM)-based nomogram and the AJCC staging system.

## RESULTS

### Patient demographics and outcomes

We retrospectively studied 2,205 patients underwent curative resection in Sun Yat-sen University Cancer Center from 2000 to 2012. Patients in the training set (n=1,470) and the internal validation set (n=735) were analyzed respectively. The mean age was 57.1±12.0 and 57.7±11.6 for training set and validation set separately. There were 1,002 men in the training set and 496 men in the validation set. 36.6% of the patients died by the time of this report. The 5-year overall survival was 55.3%. The median follow-up was 49.4 months in training set and 22.6 months in validation set. The mean number of examined lymph nodes was 20.5±12.0 and 25.8±11.8, and the mean number of positive lymph nodes was 5.8±7.7 and 6.0±7.9 in training and validation set respectively. The clinicopathologic characteristics for the training set and validation set were listed in Table 1 and the baseline characteristics for the two external validations were listed in Table 2.

**Table 1:** Characteristics of training set and validation set

	Training set (n=1470)		Validation set (n=735)	
	NO.	%	NO.	%
Age (years)				
Median	57.1±12.0		57.7±11.6	
Range	19 to 89		16 to 86	
Sex				
Male	1002	68.2	496	67.5
Female	468	31.8	239	32.5
Tumor size (cm)				
< 2	78	5.3	54	7.3
2-4	433	29.5	275	37.4
4-6	527	35.9	224	30.5
6-8	297	20.2	126	17.1
≥ 8	135	9.1	56	7.6
Tumor location				
Upper	662	45.0	324	44.1
Middle	264	18.0	113	15.4
Lower	544	37.0	298	40.5
Pathology type				
Differentiated	1110	75.5	653	88.8
Undifferentiated	360	34.5	82	11.2
Depth of invasion				
Mucosa or submucosa	122	8.3	110	15.0
Proper muscle	169	11.5	90	12.2
Subserosa	231	15.7	285	38.8
Serosa	765	52.0	210	28.6
Adjacent invasion	183	12.4	40	5.4
Positive LN (Mean±SD)	5.8±7.7		6.0±7.9	
Total LN (Mean±SD)	20.5±12.0		25.8±11.8	
≤ 15	578	39.3	158	21.5
≥ 16	892	60.7	577	78.5
mLNR (Mean±SD)	0.3±0.3		0.2±0.3	
AJCC Stage				
IA	95	6.5	73	9.9
IB	88	6.0	67	9.1
IIA	119	8.1	78	10.6
IIB	222	15.1	125	17.0
IIIA	194	13.2	113	15.4
IIIB	327	22.2	149	20.3
IIIC	425	28.9	130	17.7

Abbreviation: LN: lymph node; mLNR: metastatic lymph node ratio; AJCC: American Joint Committee on Cancer.

**Table 2:** Characteristics of validation sets

	JXCH-validation set (n=602)		SEER-validation set (n=3317)	
	NO.	%	NO.	%
Age (years)				
Median	59.0±10.7		69.0±12.0	
Range	23 to 83		20 to 90	
Sex				
Male	434	72.1	2186	65.9
Female	168	27.9	1131	34.1
Tumor size (cm)				
< 2	49	8.1	533	16.1
2-4	227	37.7	1076	32.4
4-6	182	30.2	864	26.1
6-8	116	19.3	518	15.6
≥ 8	28	4.7	326	9.8
Tumor location				
Upper	117	19.5	1157	34.9
Middle	161	26.7	1018	30.7
Lower	324	53.8	1142	34.4
Pathology type				
Differentiated	172	28.6	248	7.5
Undifferentiated	430	71.4	3069	92.5
Depth of invasion				
Mucosa or submucosa	94	15.6	707	21.3
Proper muscle	123	20.4	565	17.0
Subserosa	116	19.3	1460	44.0
Serosa	149	24.8	437	13.2
Adjacent invasion	120	19.9	148	4.5
Positive LN (Mean±SD)	4.72±8.0		2.86±5.24	
Total LN (Mean±SD)	27.65±16.1		17.77±12.56	
≤ 15	132	21.9	1744	52.6
≥ 16	470	78.1	1573	47.4
mLNR (Mean±SD)	0.19±0.32		0.17±0.25	
AJCC Stage				
IA	79	13.1	516	15.6
IB	97	16.1	370	11.2
IIA	66	11.0	462	13.9
IIB	80	13.3	619	18.7
IIIA	33	5.5	570	17.2
IIIB	107	17.8	508	15.3
IIIC	140	23.3	272	8.2

Abbreviation: LN: lymph node; mLNR: metastatic lymph node ratio; AJCC: American Joint Committee on Cancer.

### Development and validation of the nodes ratio staging system

**Figure 1:**
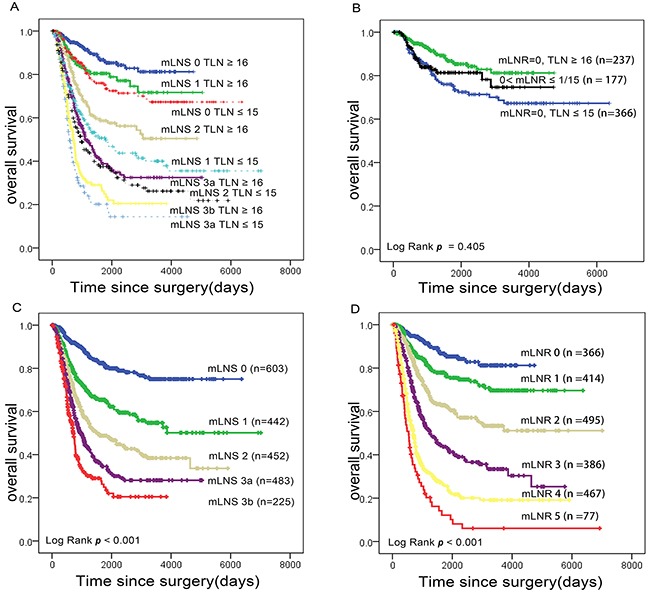
Impact of mLNS and mLNR staging on gastric cancer-related survival respectively **(A)** Overall survival according to AJCC N stage (mLNS), stratified by the number of examined nodes (≤15 and ≥16). **(B)** Overall survival of node-negative patients with 15 or less retrieved, mLNR (0 to 1/15) and node-negative patients with 16 or more retrieved. **(C)** Overall survival according to mLNS stage. **(D)** Overall survival according to mLNR stage. Abbreviation: mLNS, metastatic lymph node stage; mLNR, metastatic lymph node ratio.

Based on the SYSUCC data set, we categorized all the included patients into two groups (TLN ≥16 and TLN ≤15), Patients with examined lymph nodes ≥16 showed better prognosis than those ≤15 with respective TNM categories (p<0.001) (Figure 1A). Node-negative patients with 15 or less retrieved has no difference with mLNR (0 to 1/15) patients for overall survival (p=0.405), and node-negative patients with 16 or more exhibited significantly better survival than mLNR 0 with TLN ≤15 (p<0.001) (Figure 1B). Thus, the mLNR 1 was defined as mLNR (0 to 1/15) and node-negative patients with 15 or less retrieved, while the node-negative patients with 16 or more were classified as mLNR 0. According to X-tile, the cutoff points of 25% and 47% were used for the other patients. (Supplementary Figure 1) We also analyzed the survival of the patients by the Kaplan Meier method and survival curves, and found the survival differences within respective categories were more obvious in the TRM than that in TNM staging system (Figure 1C, 1D).

### Independent risk factors in the training set

Variables were transformed and examined to fit the Cox Proportion Hazard Regression. In the univariate analysis, age, gender, tumor location, tumor size, patho-logical type, depth of invasion (pT), N stage (mLNS), mLNR and total number of examined lymph nodes (TLN) were statistically significant prognostic factors. Significant variables were included into the multivariate analysis by the forward method. In multivariate analyses, age, tumor location, pathological type, pT and TLN were identified as the independent risk factors for overall survival (OS) (Table 3), while gender and tumor size were excluded. Both mLNS and mLNR were found to be statistically significant (p<0.001).

**Table 3:** Multivariate analysis of the training set

	Model-TNM			Model-TRM		
	HR	95% CI	*p*	HR	95% CI	*p*
Age	1.02	1.01 to 1.03	<0.001	1.02	1.01 to 1.03	<0.001
Location			<0.001			<0.001
Lower	ref			ref		
Middle	1.37	1.09 to 1.73		1.43	1.13 to 1.81	
Upper	1.57	1.31 to 1.90		1.55	1.29 to 1.86	
Pathological type	1.25	1.05 to 1.50	0.028	1.23	1.03 to 1.46	0.022
NO. of examined LNs	0.97	0.96 to 0.98	0.013			
Depth of invasion			<0.001			<0.001
Mucosa or submucosa	ref			ref		
Proper muscle	4.76	1.69 to 13.41		5.01	1.78 to 14.11	
Subserosa	6.13	2.23 to 16.86		6.11	2.22 to 16.81	
Serosa	10.84	4.00 to 29.36		10.69	3.94 to 28.98	
Adjacent invasion	15.97	5.83 to 43.75		16.18	5.90 to 44.36	
mLNR						<0.001
0				ref		
1				1.49	0.98o 2.27	
2				2.14	1.44 to 3.18	
3				3.41	2.31 to 5.03	
4				5.90	4.04 to 8.61	
5				6.56	4.19 to 10.27	
mLNS			<0.001			
0	ref					
1	1.87	1.40 to 2.49				
2	2.54	1.94 to 3.33				
3a	4.26	3.24 to 5.60				
3b	7.06	5.03 to 9.90				

Abbreviation: HR: hazard ratio; mLNR: metastatic lymph node ratio; mLNS: metastatic lymph node stage.

### Construction and validation of the nomogram

**Figure 2:**
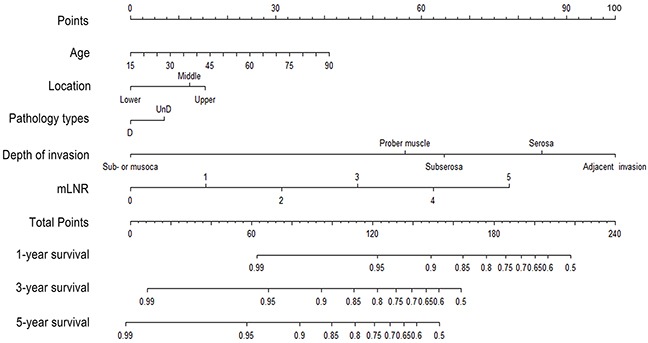
Nomogram predicting 1-year, 3-year and 5-year OS for resectable gastric cancer patients after curative resection Abbreviation: mLNR: metastatic lymph node ratio.

Two potential nomograms, TNM and TRM-based were constructed respectively based on the training set. Figure 2 shows predicting 1-year, 3-year and 5-year survival of the nomogram established based on the TRM variables. By adding up the points identified on the points scale, the nomogram can predict the likehood of 1-year, 3-year and 5-year OS for individual patient according to the total score showed in the bottom scale. The C-index for TNM-based model exhibited superior to the AJCC-TNM staging system (0.73, 95%CI: 0.69 to 0.78 vs 0.69, 95%CI: 0.65 to 0.74, p<0.001). Furthermore, when the mLNS was replaced by mLNR, the C-index of the TRM model nomogram significant increased from 0.73 to 0.76 (p<0.001). It indicated that the nomogram based mLNR was the optimal model, compared with TNM-nomogram and AJCC-TNM staging system.

**Figure 3:**
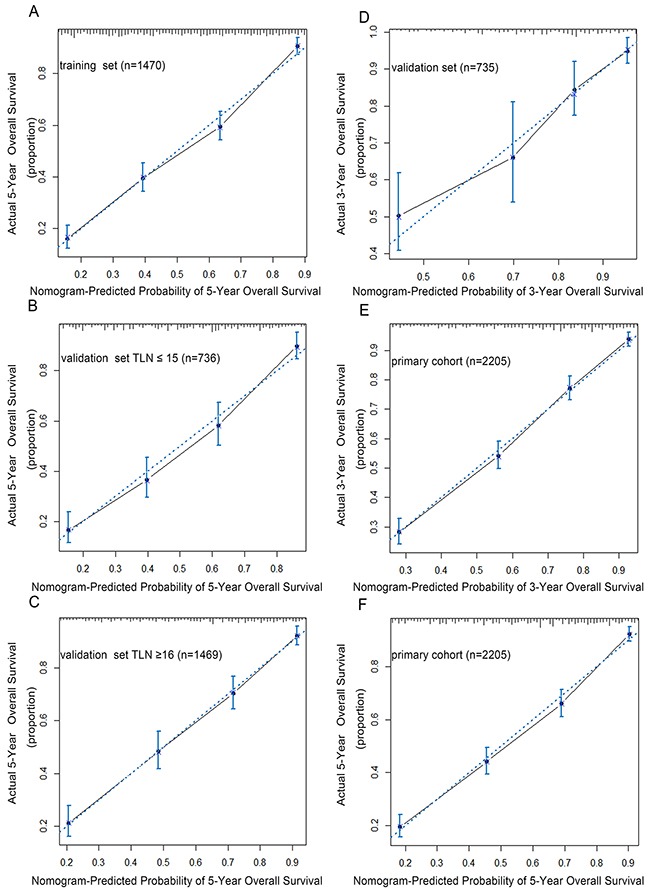
The calibration curves for predicting patients overall survival at 5-year in the training set **(A)**, validation set with TLN ≤ 15 **(B)**, validation set with TLN ≥ 16 **(C)**, validation set **(D)** and predicting overall survival at 3-year **(E)**, 5-year **(F)** in primary cohort. The X-aixs represents the nomogram-predicted survival, and the actual survival is plotted on the Y-axis. The dotted line represents the ideal correlationship between predicted and actual survival.

**Figure 4:**
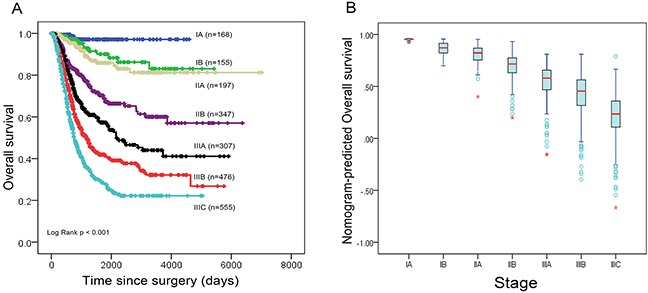
**(A)** Overall survival of primary cohort according to the 7th edition AJCC staging system; **(B)** Distribution of nomogram-predicted 5-year overall survival based on the 7th edition of AJCC staging system.

In addition, the calibration plots were separately performed by the training set, internal validation set and primary cohort. As shown in the Figure 3, the calibration plot shows that the predicted 3-year and 5-year overall survival corresponded closely with the actual survival estimated by the Kaplan-Meier method (Figure 3A, 3B, 3E, 3F). Since patients with examined lymph nodes ≥16 survives better than those ≤15 (p<0.001) (Figure 1A), we further validated our results in both TLN≥16 (n=1,469) and ≤15 (n=736) groups by using these three models (Figure 3C, 3D). Notably, in the TLN ≥16 group, the TRM-based nomogram has the higher C-index value (0.77) than TNM-based nomogram (0.75) and AJCC-TNM staging system (0.72) (p value<0.001). The results was similar in the TLN≤15 group (TRM-based nomogram, TNM-based nomogram and AJCC-TNM staging system with the C-index were 0.75, 0.73 and 0.70, respectively) (p<0.001). Figure 4A shows the 5-year survival in different stages predicted by the AJCC TNM Staging system, with no good discrimination between patients with stage IB and IIA. However, within respective TNM categories, a wide range of predicted survival could be identified by the nomogram (Figure 4B).

In the JXCH validation set, the C-index of the TRM-based nomogram was 0.76, (95%CI: 0.72 to 0.80), higher than that of the TNM-based nomogram and the 7^th^ AJCC system (0.74, 95%CI: 0.70 to 0.78 and 0.73, 95%CI: 0.69 to 0.76) (p<0.001). Consistently, TRM-based nomogram maintained the optimal discrimination both in the TLN ≥16 group (C-index: 0.74, 95%CI: 0.70 to 0.78 for TRM-based nomogram, C-index: 0.73, 95%CI: 0.68 to 0.77 for TNM-based nomogram and C-index: 0.72, 95%CI: 0.68 to 0.76 for 7^th^ AJCC system) (p<0.001) and TLN≤15 group (C-index: 0.78, 95%CI: 0.70 to 0.84 for TRM-based nomogram, C-index: 0.77, 95%CI: 0.71 to 0.84 for TNM-based nomogram and C-index: 0.76, 95%CI: 0.69 to 0.82) (p<0.001). Supplementary Figure 2 shows the calibration plots of the TRM-based nomogram.

Similarily, in the SEER validation set, TRM-based nomogram still had a superior discrimination than the other two staging systems (C-index: 0.75, 95%CI: 0.74 to 0.77 for TRM-based nomogram, C-index: 0.73, 95%CI: 0.72 to 0.75 for TNM-based nomogram and C-index: 0.70, 95%CI: 0.69 to 0.72 for the 7^th^ AJCC system) (p<0.001). Consistent results were got in the two subgroups (the TLN ≥16 group and the TLN≤15 group), the TRM-based nomogram kept the optiaml C-index value (0.74 for TLN ≥16 group and 0.76 for TLN≤15 group), higher than those of the other systems (all p value<0.001). Considering the longest follow up time of SEER data was 47 months, 5-year calibration was not accessible in our study. Supplementary Figure 3 shows 1-year and 3-year calibration plots of the TRM-based nomogram.

## DISCUSSION

In the present study, a large number of 2,205 gastric cancer patients underwent curative resection were involved to develop a nomogram based on mLNR, which could predict the survival better than the current TNM-based nomogram and AJCC TNM staging system. Especially, we firstly evaluated the significance of the nomogram in the patients with TLN ≤15 and validated the nomogram based both on eastern and western populations.

Previously, we had found that the mLNR was a better independent prognostic predictor of gastric cancer patients compared with mLNS. A staging system based on mLNR should be considered to be an alternative to the 7th AJCC TNM staging system [19, 21]. Similar results were achieved in many other studies. For example, in 2013, Ala et al performed a study about gastric cancer patients and figured out that the staging system based on mLNR was superior to TNM system [22]. Li X et al retrospectively reviewed a total 535 gastric cancer patients at different pT stages, and found that mLNR had a much better prediction ability comparable to that of pN stage in 2015 [23]. Consistently, in the current study, the nodes ratio staging system successfully stratified patients more obviously according to their survival risks, compared to the nodes staging system, which further showed that the TRM staging system worked better in prognostic prediction than the TNM staging systems.

Furthermore, we developed a nomogram based on mLNR along with other clinicopathologic parameters. In the training set, TNM-based and TRM-based nomogram were constructed respectively. We found that the TNM-based nomogram predicts survival more accurate than the AJCC TNM Staging system (C-index value: 0.73 vs 0.69, p<0.001). Interestingly, when the new factor mLNR was included in the nomogram, the TRM-based nomogram had a much higher C-index value (C-index=0.76) and predicted survival more accurately than the other two approaches. in the internal validation set, the calibration plot shows that the predicted 3-year and 5-year overall survival corresponded closely with the actual survival estimated by the Kaplan-Meier method. Additionally, external validations further indentified the discrimination of our TRM-based nomogram, it maintained the optimal C-index and calibration plot in the JXCH and SEER validation sets.

Since a least number of 15 lymph nodes was necessary for appropriate staging of gastric cancer according to the 7^th^ AJCC system, it is difficult to assess the prognosis of gastric cancer patients with insufficient nodes retrieved [5]. In fact, the significance of the nomogram has not ever been evaluated in the patients with TLN≤15. In this study, we take into consideration the influence of the TLN to the accuracy of prognostic prediction for the first time. Primary cohort and validation sets were stratified by the cutoff point (TLN ≥16 and TLN ≤15) and analyzed by the three aforementioned staging system respectively. Still, we found that the TRM-based nomogram had the much higher C-index than other models whenever in TLN ≥16 or TLN ≤15 group (p<0.001). It suggests that the discrimination power of nomogram based on mLNR is superior to the TNM-based nomogram and the 7^th^ AJCC staging system. Clearly, our study showed that a TRM-based nomogram could predict survival for gastric cancer more accurately regardless of TLN.

There may be several potential reasons for the superiority of nomogram based on the mLNR. Firstly, the number of metastatic lymph nodes is associated with the surgical and pathologic procedure and varies with the efforts and techniques of the pathologists and surgeons. Improper stage might be acquired due to the insufficient lymph nodes retrieved in surgery, leading to ‘stage migration’ [24]. Secondly, there may be the possibilities of micro-metastases in those negative lymph nodes. Patients with micro-metastases usually share a higher risk of recurrence [25]. In this study, we not only included clinicpathological variables like age, tumor location, pT, TLN and mLNS, but also the newly proposed mLNR, which remains excellent accuracy regardless of total number of the examined lymph nodes [25]. Finally, both professional doctors and gastric cancer patients could assess the individualized survival by performing such a costless and easily accessible scoring system.

Note that the most critical argument for a predictive model is the applicability. We performed our nomogram by a multi-institution method, based on both eastern and western populations. To establish a novel nomogram ignoring the influences of factors like improvements of surgical technique, nursing, medication and the quality of care in different periods, the internal validation set was not assigned by commonly used conventional random method but by the time sequence, as our prior study [26]. Actually, it is unsatisfactory to assess a nomogram by only internal validation because of the heterogeneity in data record and collection, which could be well solved by external validation. Subsequently, to justify its clinical usefulness, an external validation based on anther hospital in Jiangxi (JXCH validation set) was also performed to avoid selective bias and identify its universal applicability [27]. Mover, a high quality database, SEER database was also used for validation. Unlike common database, it is a national collaboration program by the National Cancer Institute, containing nearly 3,000,000 cases from various regions and covers 26% American population's cancer incidence and survival data, which is reliable for data quality. Surprisingly, our nomogram showed satisfactory predictive value not only in populations in China, but also in the Americans. The comprehensive validations further ascertained the applicability of our model in different populations.

Despite the satisfactory results in our studies, there are also some limitations in our studies. First, the current study involves only patients underwent curative resection, whether the results was suitable for other surgical strategies was not sure. Second, as a retrospective study, more proven significant variables such as Lauren classification, post-operative chemotherapy, radiation therapy, physical status, genomic characteristics are not available in our study, further investigations are needed in the future.

In summary, based on the training and validation sets, we analyzed the survival using three kinds of staging system separately. For the first time, we demonstrated that the TRM-based nomograms predicts the survival of gastric cancer patients more accurately than previous TNM-based nomogram and AJCC TNM staging system regardless of the number of examined lymph nodes. Given that prognosis remains uncertain and continues to be debated for gastric cancer, especially for the patients with TLN ≤15, this nomogram will be very useful when we evaluating adjuvant treatment options.

## MATERIALS AND METHODS

### Patients

We retrospectively reviewed a total number of 2,205 patients (Primary cohort) between 2000 and 2012, all of who were hospitalized in the Department of Gastropancreatic surgery, Sun Yat-sen University Cancer Center (SYSUCC), Guangzhou, China. The patients enrolled met the following criteria: patients with pathologically or histologically proven gastric cancer; no history of preoperative neo-adjuvant chemotherapy; no distant metastasis; underwent curative resection; R0 resection (No macroscopic and microscopic residual tumor); no history of other malignancies.

Additionally, Two external data sets met the aforementioned criteria (Jiangxi Provincial Cancer Hospital (JXCH) data set, from 2008 to 2013, n=602 and the Surveillance, Epidemiology, and End Results (SEER) data set, from 2010 to 2013, n=3,317) were also analyzed for validation.

### Factors

Factors like host status (age, gender), tumor characteristics (size, location, histological type, depth of invasion, number of metastatic lymph nodes, and total number of examined lymph nodes (TLN)) and follow-up data (follow up duration and survival status) were reviewed in our data set. The tumor size was measured as the widest diameter and grouped by the cutoff points of 2cm, 4cm, 6cm and 8cm. The tumor location was defined as upper third, middle third and lower third based on the main center of the lesion. As for the histological type, papillary, tubular adenocarcinoma and mucinous adenocarcinoma were included in the differentiated type, while signet ring cell carcinoma and small cell carcinoma were defined as the undifferentiated type. The classification of the depth of invasion and lymph node metastasis (mLNS) were performed according to the 7th AJCC TNM staging system [5].

### Follow up

Patients were followed up by post-operative clinical and laboratory examinations per 3 months during the first 2 years, per 6 months from the third year to the fifth year and annually until he/she died. The follow-up duration was defined as the interval between the surgery and last follow up, and overall survival time was defined as the time between the surgery and all-cause death.

### Development of the nodes ratio staging system

For the training set, we classified the node-negative patients with 15 or less as mLNR 1, and the node-negative patients with 16 or more as mLNR 0 [5]. We categorized the node-positive patients into 5 groups according to the following criteria: mLNR 1: 0<mLNR≤1/15; mLNR 2: 1/15<mLNR≤25%; mLNR 3: 25%<mLNR≤47%; mLNR 4: 47%<mLNR≤99%; mLNR 5: mLNR= 100%. The cutoff points were identified by the X-tile software version 3.6.1 (Yale University School of Medicine, New Haven, CT, USA) [28]. Thus, a new TRM staging system was constructed based on our mLNR stage.

### Construction of the nomogram

For the development and internal validation of the nomogram, the 2,205 population was divided in two groups. Patients who underwent curative resection before December 31, 2008 were assigned to the training set (n=1,470) and patients who underwent surgery between January 1, 2009 and December 31, 2012 (n=735) were selected as the internal validation set. By the Multivariate Cox Proportional Hazards Regression analysis of the training set, the independent risk factors were identified. And then, nomogram based on the independent risk factors was constructed. In this study, we built TNM-based and TRM-based nomogram separately. The former nomogram was constructed mainly based on the number of the examined lymph nodes, which is the same as the prior studies [11, 13]. However, the latter based on mLNR, is a novel model.

### Validation of the nomogram

The performance of the nomogram was evaluated by discrimination and calibration using internal (SYSUCC, n=735) and two external (JXCH validation set, n=602 and SEER validation set, n=3,317) validation sets. Regarding discrimination, Harrell's C-index was used, which is appropriate for censored data and similar to the area under the receiver operating characteristic (ROC) curve [29]. C-index provides the probability between the observed and predicted OS. Generally, the C-index acts as a measure of the accuracy of a nomogram, and a value more than 0.75 usually indicates relatively good discrimination (the closer it is to 1.0, the more accurate it is) [30]. For calibration, the data was divided into several groups based on the probabilities calculated by the nomogram predictive model. Subsequently, predicted probabilities produced by the nomogram was compared with actual probabilities by the Kaplan Meier method. H-L chi-square statistic and bootstrapping correction were used for this purpose.

P value<0.05 was considered to be statistically significant. All analyses were performed by the software statistical package for social sciences version 20.0 (SPSS, Chicago, IL) and the package of *rms* in R software version 3.13 (http://www.r-project.org/).

## SUPPLEMENTARY FIGURES



## Abbreviations

mLNR: metastatic lymph nodes ratio
mLNS: metastatic lymph nodes stage
AJCC: American Joint Committee on Cancer
TLN: total number of examined lymph nodes

## References

[1] Jemal A, Bray F, Center MM, Ferlay J, Ward E, Forman D (2011). Global cancer statistics. CA Cancer J Clin.

[2] Bertuccio P, Chatenoud L, Levi F, Praud D, Ferlay J, Negri E, Malvezzi M, La Vecchia C (2009). Recent patterns in gastric cancer: a global overview. Int J Cancer.

[3] DeSantis CE, Lin CC, Mariotto AB, Siegel RL, Stein KD, Kramer JL, Alteri R, Robbins AS, Jemal A (2014). Cancer treatment and survivorship statistics, 2014. CA Cancer J Clin.

[4] Songun I, Putter H, Kranenbarg EM, Sasako M, van de Velde CJ (2010). Surgical treatment of gastric cancer: 15-year follow-up results of the randomised nationwide Dutch D1D2 trial. Lancet Oncol.

[5] Washington K (2010). edition of the AJCC cancer staging manual: stomach. Ann Surg Oncol.

[6] Wang Y, Li J, Xia Y, Gong R, Wang K, Yan Z, Wan X, Liu G, Wu D, Shi L, Lau W, Wu M, Shen F (2013). Prognostic nomogram for intrahepatic cholangiocarcinoma after partial hepatectomy. J Clin Oncol.

[7] Xie D, Marks R, Zhang M, Jiang G, Jatoi A, Garces YI, Mansfield A, Molina J, Yang P (2015). Nomograms predict overall survival for patients with small-cell lung cancer incorporating pretreatment peripheral blood markers. J Thorac Oncol.

[8] Elshafei A, Kovac E, Dhar N, Levy D, Polascik T, Mouraviev V, Yu C, Jones JS (2015). A pretreatment nomogram for prediction of biochemical failure after primary cryoablation of the prostate. Prostate.

[9] Kawai K, Ishihara S, Yamaguchi H, Sunami E, Kitayama J, Miyata H, Watanabe T (2015). Nomogram prediction of metachronous colorectal neoplasms in patients with colorectal cancer. Ann Surg.

[10] Frasson M, Flor-Lorente B, Rodriguez JL, Granero-Castro P, Hervas D, Alvarez Rico MA, Brao MJ, Sanchez Gonzalez JM, Garcia-Granero E (2015). Risk factors for anastomotic leak after colon resection for cancer: multivariate analysis and nomogram from a multicentric, prospective, national study with 3193 patients. Ann Surg.

[11] Kattan MW, Karpeh MS, Mazumdar M, Brennan MF (2003). Postoperative nomogram for disease-specific survival after an R0 resection for gastric carcinoma. J Clin Oncol.

[12] Han DS, Suh YS, Kong SH, Lee HJ, Choi Y, Aikou S, Sano T, Park BJ, Kim WH, Yang HK (2012). Nomogram predicting long-term survival after d2 gastrectomy for gastric cancer. J Clin Oncol.

[13] Eom BW, Ryu KW, Nam BH, Park Y, Lee HJ, Kim MC, Cho GS, Kim CY, Ryu SW, Shin DW, Hyung WJ, Lee JH (2015). Survival nomogram for curatively resected Korean gastric cancer patients: multicenter retrospective analysis with external validation. PLoS One.

[14] Hirabayashi S, Kosugi S, Isobe Y, Nashimoto A, Oda I, Hayashi K, Miyashiro I, Tsujitani S, Kodera Y, Seto Y, Furukawa H, Ono H, Tanabe S (2014). Development and external validation of a nomogram for overall survival after curative resection in serosa-negative, locally advanced gastric cancer. Ann Oncol.

[15] Kim JH, Kim HS, Seo WY, Nam CM, Kim KY, Jeung HC, Lai JF, Chung HC, Noh SH, Rha SY (2012). External validation of nomogram for the prediction of recurrence after curative resection in early gastric cancer. Ann Oncol.

[16] Dicken BJ, Bigam DL, Cass C, Mackey JR, Joy AA, Hamilton SM (2005). Gastric adenocarcinoma: review and considerations for future directions. Ann Surg.

[17] Marchet A, Mocellin S, Ambrosi A, Morgagni P, Garcea D, Marrelli D, Roviello F, de Manzoni G, Minicozzi A, Natalini G, De Santis F, Baiocchi L, Coniglio A, Nitti D (2007). The ratio between metastatic and examined lymph nodes (N ratio) is an independent prognostic factor in gastric cancer regardless of the type of lymphadenectomy: results from an Italian multicentric study in 1853 patients. Ann Surg.

[18] Sun Z, Zhu GL, Lu C, Guo PT, Huang BJ, Li K, Xu Y, Li DM, Wang ZN, Xu HM (2009). The impact of N-ratio in minimizing stage migration phenomenon in gastric cancer patients with insufficient number or level of lymph node retrieved: results from a Chinese mono-institutional study in 2159 patients. Ann Oncol.

[19] Wang W, Xu DZ, Li YF, Guan YX, Sun XW, Chen YB, Kesari R, Huang CY, Li W, Zhan YQ, Zhou ZW (2011). Tumor-ratio-metastasis staging system as an alternative to the 7th edition UICC TNM system in gastric cancer after D2 resection--results of a single-institution study of 1343 Chinese patients. Ann Oncol.

[20] Wang J, Dang P, Raut CP, Pandalai PK, Maduekwe UN, Rattner DW, Lauwers GY, Yoon SS (2012). Comparison of a lymph node ratio-based staging system with the 7th AJCC system for gastric cancer: analysis of 18,043 patients from the SEER database. Ann Surg.

[21] Xu DZ, Geng QR, Long ZJ, Zhan YQ, Li W, Zhou ZW, Chen YB, Sun XW, Chen G, Liu Q (2009). Positive lymph node ratio is an independent prognostic factor in gastric cancer after d2 resection regardless of the examined number of lymph nodes. Ann Surg Oncol.

[22] Alatengbaolide LD, Li Y, Xu H, Chen J, Wang B, Liu C, Lu P (2013). Lymph node ratio is an independent prognostic factor in gastric cancer after curative resection (R0) regardless of the examined number of lymph nodes. Am J Clin Oncol.

[23] Li X, Liu Y, Cao B, Liu B, Bai T, Li X, Mei L, Che X (2015). Metastatic lymph node ratio and prognosis of gastric cancer at different pT stages. Hepatogastroenterology.

[24] Feinstein AR, Sosin DM, Wells CK (1985). The Will Rogers phenomenon. Stage migration and new diagnostic techniques as a source of misleading statistics for survival in cancer. N Engl J Med.

[25] Xu D, Huang Y, Geng Q, Guan Y, Li Y, Wang W, Yuan S, Sun X, Chen Y, Li W, Zhou Z, Zhan Y (2012). Effect of lymph node number on survival of patients with lymph node-negative gastric cancer according to the 7th edition UICC TNM system. PLoS One.

[26] Liu J, Geng Q, Liu Z, Chen S, Guo J, Kong P, Chen Y, Li W, Zhou Z, Sun X, Zhan Y, Xu D (2016). Development and external validation of a prognostic nomogram for gastric cancer using the national cancer registry. Oncotarget.

[27] Iasonos A, Schrag D, Raj GV, Panageas KS (2008). How to build and interpret a nomogram for cancer prognosis. J Clin Oncol.

[28] Camp RL, Dolled-Filhart M, Rimm DL (2004). X-tile: a new bio-informatics tool for biomarker assessment and outcome-based cut-point optimization. Clin Cancer Res.

[29] Harrell FE, Lee KL, Mark DB (1996). Multivariable prognostic models: issues in developing models, evaluating assumptions and adequacy, and measuring and reducing errors. Stat Med.

[30] Harrell FE, Califf RM, Pryor DB, Lee KL, Rosati RA (1982). Evaluating the yield of medical tests. JAMA.

