# Supplementary material for: Lymph nodes ratio based nomogram predicts survival of resectable gastric cancer regardless of the number of examined lymph nodes
Source: Oncotarget. 2017 Apr 20;8(28):45585–96. doi: 10.18632/oncotarget.17276 (PMC5542210; doi:10.18632/oncotarget.17276)
Supplement: Supplementary file 1 [file oncotarget-08-45585-s001.pdf]

# Lymph nodes ratio based nomogram predicts survival of resectable gastric cancer regardless of the number of examined lymph nodes

## SUPPLEMENTARY FIGURES

mLNR stage

mLNR 0: mLNR = 0 with TLN ≥ 16;
mLNR 1: mLNR = 0 with TLN ≤ 15,
0 < mLNR ≤ 1/15;
mLNR 2: 1/15 < mLNR ≤ 25%;
mLNR 3: 25% < mLNR ≤ 47%;
mLNR 4: 48% < mLNR ≤ 99%;
mLNR 5:mLNR = 100%;

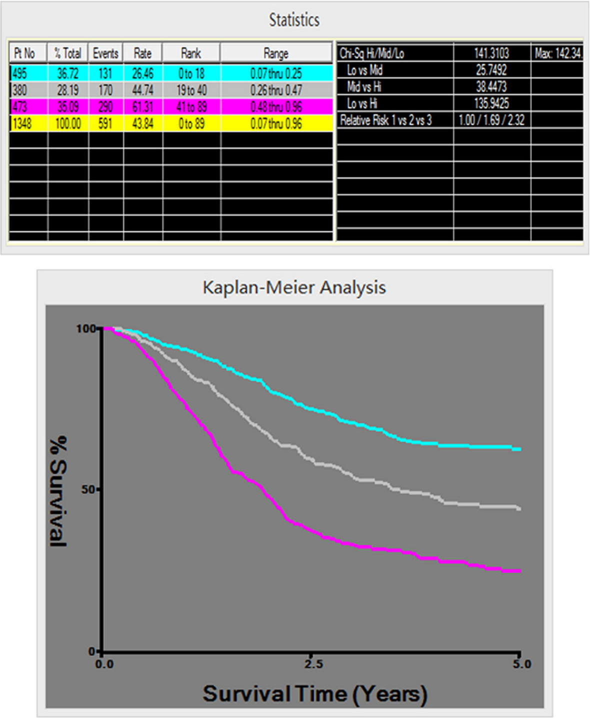


**Supplementary Figure 1: The optimal cutoff points stratified by the outcome of the primary cohort according to the X-tile plots.**

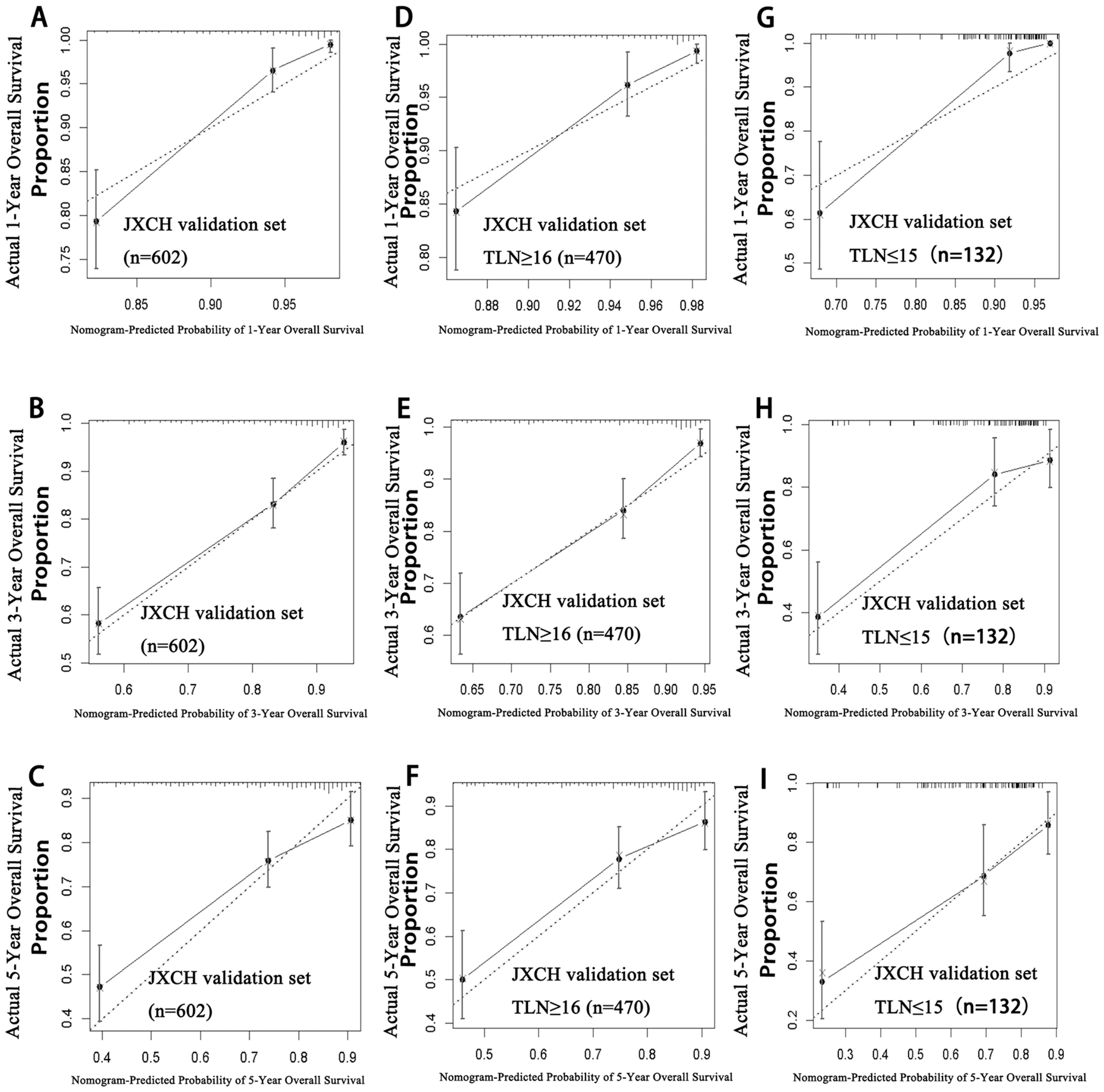


**Supplementary Figure 2: The calibration curves for predicting patients overall survival at 1-year (A), 3-year (B), 5-year in the JXCH validation set (C); predicting overall survival at 1-year (D), 3-year (E), 5-year (F) in in the JXCH validation set with TLN ≥ 16 and predicting overall survival at 1-year (G), 3-year (H), 5-year (I) in JXCH validation set with TLN ≤ 15.** The X-aixs represents the nomogram-predicted survival, and the actual survival is plotted on the Y-axis. The dotted line represents the ideal correlationship between predicted and actual survival.

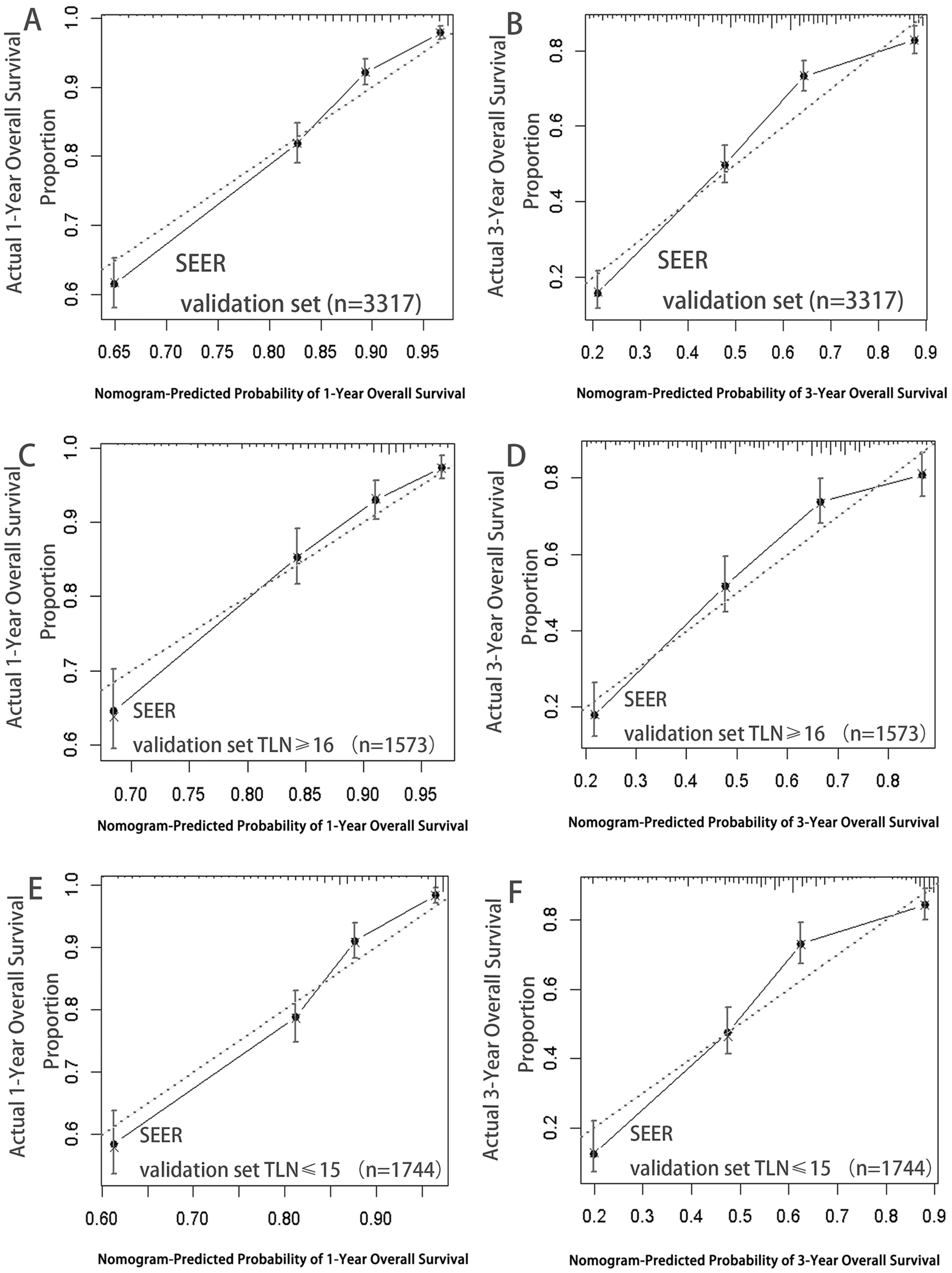


**Supplementary Figure 3: The calibration curves for predicting patients overall survival at 1-year (A), 3-year (B) in the SEER validation set; predicting overall survival at 1-year (C), 3-year (D) in in the SEER validation set with TLN ≥ 16 and predicting overall survival at 1-year (E), 3-year (F) in SEER validation set with TLN ≤ 15.**
